# Pupillary Light Reflex Induced by Two-Photon Vision

**DOI:** 10.1167/iovs.62.15.23

**Published:** 2021-12-22

**Authors:** Agnieszka Zielińska, Piotr Ciąćka, Maciej Szkulmowski, Katarzyna Komar

**Affiliations:** 1 Institute of Physics, Faculty of Physics, Astronomy and Informatics, Nicolaus Copernicus University in Toruń, Toruń, Poland; 2 International Centre for Translational Eye Research, Warsaw, Poland; 3 Department of Physical Chemistry of Biological Systems, Institute of Physical Chemistry, Polish Academy of Sciences, Kasprzaka 44/52, 01-224 Warsaw, Poland

**Keywords:** two-photon vision, pupillary light reflex, perception of light

## Abstract

**Purpose:**

Two-photon vision relies on the perception of pulsed infrared light due to two-photon absorption in visual pigments. This study aimed to measure human pupil reaction caused by a two-photon 1040-nm stimulus and compare it with pupil responses elicited by 520-nm stimuli of similar color.

**Methods:**

Pupillary light reflex (PLR) was induced on 14 dark-adapted healthy subjects. Three types of fovea-centered stimuli of 3.5° diameter were tested: spirals formed by fast scanning 1040-nm (infrared [IR] laser) or 520-nm (visible [VIS] laser) laser beams and uniformly filled circle created by 520-nm LED (VIS light-emitting diode [LED]). The power of visible stimuli was determined with a dedicated procedure to obtain the same perceived brightness equivalent as for 800 µW used for two-photon stimulation.

**Results:**

The minimum pupil diameter for IR laser was 88% ± 10% of baseline, significantly larger than for both VIS stimuli: 74% ± 10% (laser) and 69% ± 9% (LED). Mean constriction velocity and time to maximum constriction had significantly smaller values for IR than for both VIS stimuli. Latency times were similar for IR and VIS lasers and slightly smaller for VIS LED.

**Conclusions:**

The two-photon stimulus caused a considerably weaker pupil reaction than one-photon stimuli of the same shape, brightness, and similar color. The smaller pupil response may be due to weaker two-photon stimulation of rods relative to cones as previously observed for two-photon vision. Contrary to normal vision, in a two-photon process the stray light is not perceived, which might reduce the number of stimulated photoreceptors and further weaken the PLR.

Due to pupillary light reflex (PLR), the human eye adjusts the amount of light reaching the retina, thus regulating the illumination of the photoreceptors and keeping them from being saturated.^1^ The reflex is a valuable part of the standard neurological examination because it reflects the functioning of the nervous system. The PLR measurement is a well-established method in the management and prognosis of patients with acute brain injuries,^2^ idiopathic intracranial hypertension,^3^ early diagnostic of inner neuroretina changes in patients with diabetes,^4^ glaucoma,^5^^–^^7^ subclinical stages of neurodegenerative disorders such as Alzheimer's or Parkinson's disease,^8^ or in the screening of neurodevelopmental disorders in children.^9^ The PLR could also be an indicator of parasympathetic activity by testing its relationship with daily-life fatigue^10^ and a marker of both central sympathetic and parasympathetic balance in clinical studies of depression.^11^ A part of clinical diagnostics for many years, the PLR still finds new clinical applications.^12^ For example, chromatic pupilloperimetry may potentially be used for objective noninvasive assessment of rod and cone cell function in different locations of the retina.^13^^,^^14^

The magnitude of the PLR generally follows the eye's spectral sensitivity, with a maximum for green color under photopic conditions and with a blue shift under dark adaptation.^1^^,^^15^ Recently, investigations of pupil response dependence on stimulation wavelength were motivated by the discovery of intrinsically photosensitive retinal ganglion cells (ipRGCs).^16^^–^^20^ Registered pupil reactions for red light (600 nm^21^ and 650 nm^19^) were generally weaker than for green and blue. Infrared light around 1000 nm is not considered to trigger the PLR because this spectral region is not covered by the luminous spectral efficiency function, *V*(λ).^22^ However, it was recently found that short-pulsed light of this wavelength can stimulate the human visual system and is then perceived as visible light of a color close to half the wavelength applied for the stimulation.^23^ The effect is caused by two-photon absorption occurring in visual pigments; thus, it is referred to as two-photon vision.^24^ To the best of our knowledge, there is no study reporting the PLR following infrared light stimulation perceived in this way.

Studies of two-photon vision are a new research field; devices and techniques for quantifying this phenomenon are currently being developed.^24^^–^^27^ Highly localized stimulation of the retina offered by the two-photon process could benefit novel devices for ophthalmic diagnosis. Due to better penetration through opaque media, infrared light might be employed in patients suffering from cataract.^24^^,^^28^ The two-photon vision microperimetry was also successfully applied for testing patients with diabetic retinopathy^29^ and AMD.^30^ A natural variable aperture (i.e., pupil) is a crucial factor of an ophthalmic system. Therefore, whether and how the pupils react to the two-photon stimulation are essential questions in developing novel eye diagnostic modalities based on two-photon vision.

Classical psychophysical methods of measuring visual perception rely on feedback from the tested subjects; however, the PLR, upon two-photon stimulation, could serve as a relatively objective measure of a psychophysical response. It could also complement two-photon perimetry with the information on pupil reaction upon two-photon stimulus.^31^ Pupil campimetry^32^^,^^33^ or chromatic pupilloperimetry,^34^^,^^35^ in which a small area of the retina is stimulated to determine if and how the subject sees, could also be adapted for two-photon vision.

The present study aimed to establish whether and how a human pupil reacts to two-photon infrared stimulation. The two-photon–induced PLRs (1040 nm) were registered and compared with PLRs following one-photon stimuli (520 nm) of similar color. The brightness adjustment test for 520 nm and 1040 nm stimuli was conducted to obtain the same perceived intensity. To quantitatively compare the effect of two-photon stimulation with the reaction of the visual system to typical, one-photon stimuli, four PLR parameters were determined: minimum pupil diameter, latency time, mean constriction velocity, and time to maximum constriction.^36^

## Methods

### The Optical System

**Figure 1.**
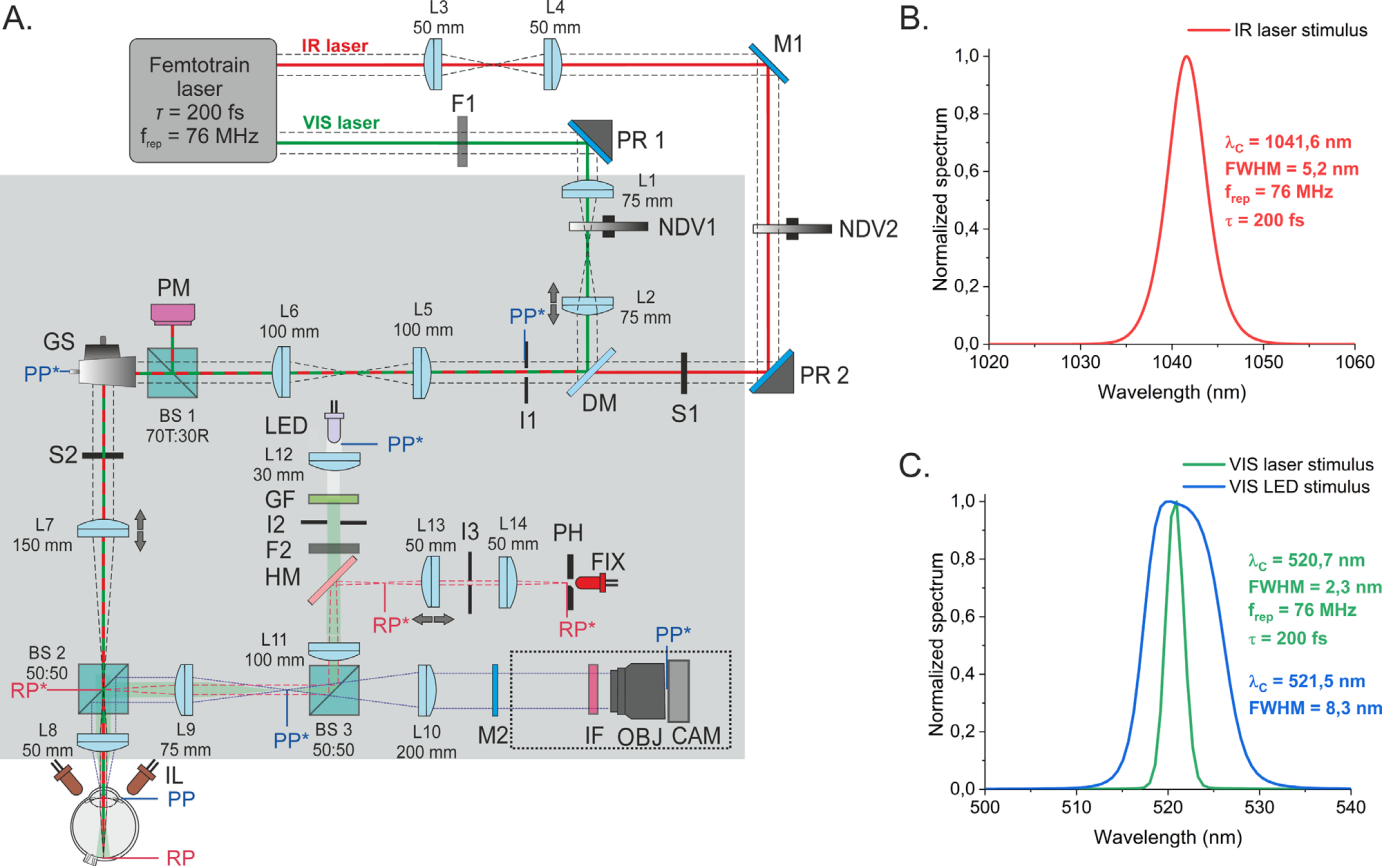
(**A**) The optical system for two-photon and visible PLR experiments. BS1–BS3, beamsplitters; CAM, camera (Thorlabs DCC 1545M); DM, dichroic mirror (T800 LPXRXT; Chroma Technology, Bellows Falls, VT, USA); F1, F2, neutral density filters; FIX, fixation LED (λ_C_ = 631 nm, full width at half maximum [FWHM] = 15 nm); GF, green filter (Thorlabs FB 520-10); GS, galvanometric scanners (Cambridge Technology, Bedford, MA, USA); HM, hot mirror; I1–I3, regulated irises; IF, infrared bandpass filter (Thorlabs FB940-10); IL, illuminator (λ_C_ = 940 nm, FWHM = 52 nm), L1–L14, lenses; LED, white LED (Thorlabs MWWHL3); M1 and M2, flat mirrors; NDV1 and NDV2, variable neutral-density filters; OBJ, objective (Thorlabs MVL35M32); PH, pinhole (100 µm); PM, power meter (Thorlabs S130C); PR1 and PR2, periscopes; S1 and S2, shutters; PP, pupil plane; PP*, conjugated pupil plane; RP, retinal plane; RP*, conjugated retinal plane. All lenses in the common path of the stimulating beams have antireflection coatings (type AB, range, 400–1100 nm) to minimize reflections from the lens surfaces for both stimulating beams. The dotted rectangle surrounds part of the optical path mounted perpendicular to the plane of the breadboard (after reflection of M2). The *gray* *rectangle* indicates part of the system placed on the breadboard (600 mm × 900 mm) and covered by a black optical enclosure. (**B**) Spectrum of the IR laser. (**C**) Spectra of the VIS laser and VIS LED.

A schematic of our custom-developed optical system is presented in Figure 1A. Both stimulating laser beams—visible (VIS; 520 nm) (Fig. 1B) and infrared (IR; 1040 nm) (Fig. 1C)—were delivered to the eye from the femtosecond laser (femtoTrain HighQ Laser; Spectra-Physics, Milpitas, CA, USA) by a galvanometric scanner. Two motorized variable neutral-density filters allowed regulation of the intensity of both stimuli. The power meter (PM) performed continuous monitoring of the power. Shutters S1 and S2 and the 4.0 neutral density filter F1 were used to block beams to the eye alternately during stimulation and PM measurements. The refraction error of the subject's eye was compensated by changing the position of motorized lenses: the L7 for the infrared (IR) beam and, independently, the L2 for the visible (VIS) beam. Diameters (1/e^2^) of both stimulating beams measured at the pupil plane were similar: 0.69 mm and 0.74 mm for the VIS and IR beams, respectively.

Directly before each measurement session, a beam power calibration was performed. An additional sensor was placed at the pupil plane, and readings of both sensors—the PM in the system (Fig. 1) and the external one—were recorded to obtain several values of each laser separately. This allowed for determination of the actual calibration factor for each stimulating beam at the output of the system. The calibration was performed without the F1 filter for the visible beam, the transmission of which for 520 nm was specified by the manufacturer (0.01171%, NE40A-A; Thorlabs, Newton, NJ, USA). The VIS power values at the pupil plane with this filter were minimal (only a few hundred picowatts), which would significantly reduce the accuracy of the power measurement. During testing, VIS beam power measurements by the PM located inside the system were also performed without the F1 filter and after closing shutter S2.

**Figure 2.**
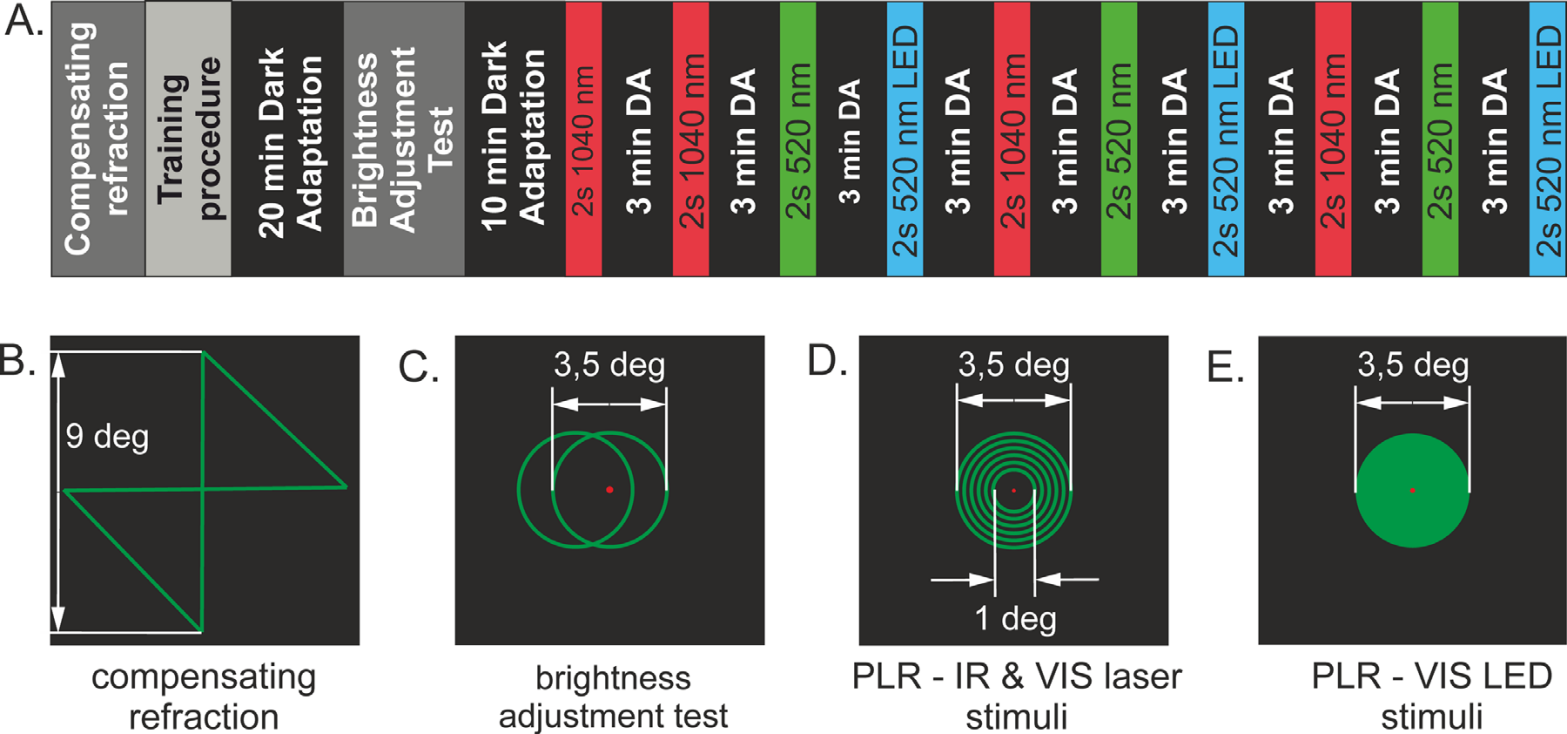
(**A**) The course of PLR measurements. The first PLR measurement induced by the 1040-nm stimulus was rejected from analysis. The stimuli shapes used during the following stages were (**B**) compensating refraction, (**C**) brightness adjustment test, (**D**) PLR following laser-based stimuli, and (**E**) PLR following LED stimulation.

The white light-emitting diode (LED) with a green filter (520 nm) provided the filled-circle stimulus (Fig. 2E) in the Maxwellian view illumination.^37^ The diameter of the light source image at the pupil plane was 3.25 mm, below the minimal registered size of subjects’ pupils. The power of LED at the system output was measured by placing an additional sensor at the pupil plane after removing filter F2 of known transmission (a set of Thorlabs filters, NE 10A-A and NE 20A-A; common transmission, 0.125%). The patient's pupil center was kept at the optical path of the system and the focal distance from the last lens through a motorized chinrest. The eye position was stabilized during the tests with a small (approximately 5 arcmin) red (631 nm) fixation point placed centrally in the patient's field of view. The movable L3 lens allowed for manual refraction error correction in the fixation path. The monochrome complementary metal oxide semiconductor (CMOS) camera with objective was used to register pupil images. The images (512 × 512 pixels) were collected at 83 frames per second, with one pixel corresponding to 18 µm at the pupil plane. The dedicated software developed in LabVIEW (National Instruments, Austin, TX, USA) fully automated operation of the system.

### Measurement Procedure

Measurements were done between 9 AM and 4 PM under scotopic conditions (<0.1 lux) at the Laboratory of Applied Biophotonics, Institute of Physics, Nicolaus Copernicus University in Toruń. The measurement procedure is shown in Figure 2A. First, in a dimly lit room (<2 lux), the subject moved lenses L7 and L2 to achieve the best focusing of, respectively, the IR and VIS stimuli (Fig. 2B). Then the subject was trained to properly perform the brightness adjustment test, described in detail further on.

Next, the subject was dark-adapted for 20 minutes with eyes covered by eye patches. Afterward, one eye preferred by the subject was uncovered and stimulated. During the brightness adjustment test (performed once), the two laser stimuli (Fig. 2C) were simultaneously projected onto the subject's retina. The IR stimulus was centered but the VIS laser stimulus was shifted by 50 arcmin left and 25 arcmin up. The subject's task was to adjust the intensity of the VIS laser stimulus to match the perceived brightness of the IR stimulus. The adjustments were made for six powers of IR laser stimulus (at the pupil plane) consecutively set by the operating software from 100 to 800 µW. This procedure delivered a dataset allowing determination of whether the mechanism of IR laser perception is nonlinear, similar to the method described in Ruminski et al.^24^ After 10 minutes of the re-adaptation period, PLR measurements following three types of stimulation centered at the fovea were performed. The spiral IR and VIS stimuli (Fig. 2D) consisted of 15 coils, and the retina were scanned with laser beams at a frame repetition rate 100 Hz. The power of the IR laser beam during tests was 800 µW at the pupil plane. As determined by the brightness adjustment procedure, the power of the VIS laser stimulus was slightly different for each subject. Mean VIS power (±1 SD) across all subjects was 266 ± 58 pW at the pupil plane. The third stimulus was a filled circle formed by a VIS LED (Fig. 2E) with the same power at the pupil plane as for the VIS laser stimulus. The radiometric and photometric quantities characterizing all stimuli measured at the pupil of the eye are provided in the Table. The pupil images were recorded for 72 seconds (the acquisition began 10 seconds before the start of the stimulus and ended 60 seconds after the end of stimulation; the stimulus lasted 2 seconds). The subject's task was to gaze on the fixation dot and avoid blinking during the critical test time (i.e., few seconds before and after stimulus presentation). Between the recording sessions, the subject could move her or his eyes freely or keep them closed. The first PLR was always rejected, in agreement with Kelbsch et al.^36^

**Table:** Radiometric and Photometric Quantities at Eye's Pupil for IR Laser, VIS Laser, and LED

Quantity at Pupil Plane	IR Laser	VIS Laser^*^	LED^*^
Total power	800 µW	266 pW	266 pW
Irradiance	2.1 mW/cm^2^	691 pW/cm^2^	691 pW/cm^2^
Log photon flux (log PHOT/cm^2^⋅s)	16.04	9.26	9.26
Photopic illuminance (photopic lux)	N/A	3.3 × 10^−3^	3.4 × 10^−3^
Scotopic illuminance (scotopic lux)	N/A	10.6 × 10^−3^	10.2 × 10^−3^
α-Opic equivalent daylight (D65) illuminance (α-opic lux)^†^			
S-cone-opic illuminance (S-cone-opic lux)	N/A	0.1 × 10^−3^	0.1 × 10^−3^
Melanopic illuminance (melanopic lux)	N/A	3.5 × 10^−3^	3.4 × 10^−3^
Rhodopic illuminance (rhodopic lux)	N/A	4.3 × 10^−3^	4.2 × 10^−3^
M-cone-opic illuminance (M-cone-opic lux)	N/A	4.1 × 10^−3^	4.2 × 10^−3^
L-cone-opic illuminance (L-cone-opic lux)	N/A	2.8 × 10^−3^	2.9 × 10^−3^

NA, not available.

* All measurements for visible sources at the system's output were performed after removing filters F1 and F2 (see Fig. 1); the exact values given here were calculated based on transmission of these filters provided by manufacturer.

† Calculated basing on spectral power distribution measured at pupil plane and International Standard CIE S 026/E:2018 (CIE System for Metrology of Optical Radiation for ipRGC-Influenced Responses to Light).

### Participants and Laser Safety Levels

The subject group was comprised of 14 healthy Caucasian subjects (seven female, seven male) ages 20 to 42 years (mean, 30.7 years; SD, 7.9 years). Three authors participated in the experiments (AZ as P9, MS as P15, and KK as P13). Among the remaining participants were six members of the Laboratory of Applied Biophotonics, Institute of Physics, Nicolaus Copernicus University in Toruń. The subjects did not report any visual problems; the mean refractive error of participants was –0.5 diopter (D), with SD = 1.0 D. The participants had normal color vision, as determined by the Ishihara test and the D-15 dichotomous test. Pupil dilation was not used.

The study protocol adhered to the tenets of the Declaration of Helsinki and was approved by the Ethics Committee of the Nicolaus Copernicus University in Toruń. Participants were informed of the nature of the experiment and the potential risks involved and signed a consent form. All tests were conducted in compliance with the Polish safety standard PN EN 60825-1:2014 and standard ANSI Z136.1-2014.

Although the laser beams continuously scanned the retina during our experiments, we complied with the stricter safety levels calculated for stationary beams. The maximum permissible radiant powers (MPΦs) calculated for 3-minute exposures of the immobilized eye for pulsed laser beams of wavelengths 1040 nm and 520 nm (pulse width, 200 fs; repetition frequency, 76 MHz) are 905 µW and 199 nW, respectively.^38^^,^^39^ In our procedure, the initial focusing of the stimuli took 1 to 2 minutes and was performed for an IR laser of power about 150 µW; the brightness adjustment test for a single power value was ∼1 minute, and each PLR stimulus lasted 2 seconds. During the periods between PLR registrations and brightness adjustment for a single power, the beams were closed with shutters S1 and S2. The highest power of the 1040-nm beam (800 µW) was used for only one of the six powers tested in the brightness adjustment and for PLR registration. The power of the 520-nm beam (some hundreds of picowatts) was three orders of magnitude lower than the calculated MPΦ. During the brightness adjustment procedure, both laser stimuli were displayed simultaneously but, due to scanning, practically never at the same retinal location.

### PLR Data Processing

**Figure 3.**
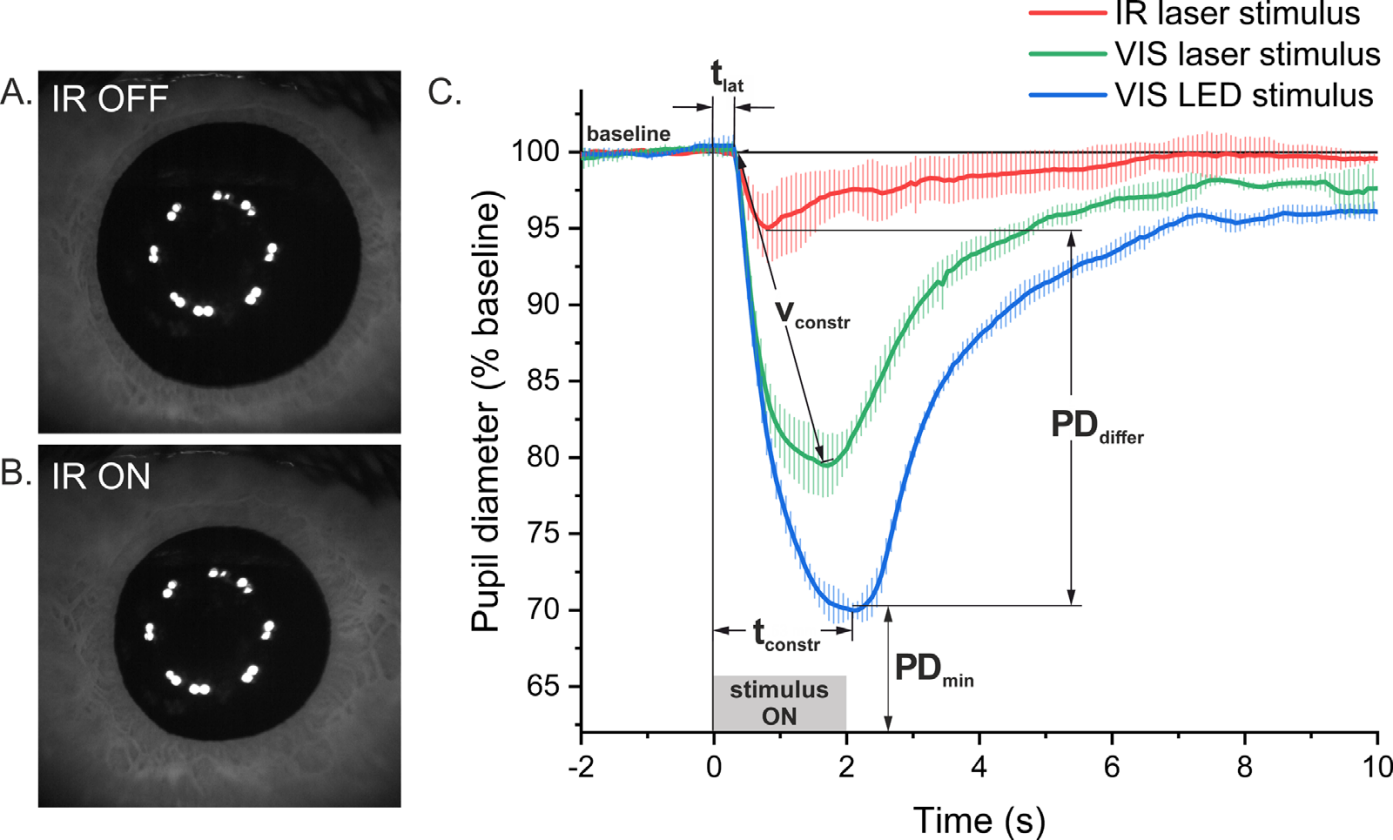
Typical images of pupil obtained with the IR laser stimulus switched off (**A**) and switched on (**B**). A *dotted ring* within the pupil image was formed by the reflections of the illuminating diodes on the cornea. (**C**) Average PLR curves of a representative subject (P2); the absolute pupil diameter data (in mm) are shown in Supplementary Figure S2. *Error bars* indicate the standard deviations of three trials. The parameters of the PLR are specified as follows: *t*_lat_, latency time (s); *t*_constr_, time of maximum constriction (s); *v*_constr_, mean constriction velocity (mm/s); *PD*_min_, minimum pupil diameter relative to baseline (%); *PD*_differ_, difference in minimum pupil diameter between the IR laser and VIS LED stimuli (%).

Blinks and other artifacts on the obtained pupil images were eliminated automatically with dedicated LabVIEW software during postprocessing. Typical pupil images are shown in Figures 3A and 3B. The pupil diameter for each recorded image was determined with separate software prepared in Python, described elsewhere.^40^ However, this algorithm could not properly define pupil diameter when the pupil was smaller or similar to the diameter of the dotted ring formed by the reflecting illuminating diodes on the cornea. For those cases, we used an additional program developed in LabVIEW to fit a circle to a pupil based on three points on a detected edge along the manually indicated lines (Supplementary Fig. S1). The numerical data obtained were further analyzed in Origin to determine the PLR parameters indicated in Figure 3C. Baseline pupil diameter (a 100% value in Fig. 3C) was calculated as a mean diameter from the period of 2 seconds before the stimulus. Relative pupil diameter at any given time *t* is related to baseline by the following formula:

(1) $$\;\;\;\begin{array}{c} \mathrm{Relative}\;\mathrm{pupil}\;\mathrm{diameter}\;\left(t\right)=\frac{\mathrm{Absolute}\;\mathrm{pupil}\;\mathrm{diameter}\;(t)}{\mathrm{Baseline}\;\mathrm{pupil}\;\mathrm{diameter}}\times 100\% \end{array}$$

Latency time (*t*_lat_) was found on the basis of velocity and acceleration analysis described in Bergamin and Kardon.^41^ Minimum pupil diameter (*PD*_min_) for a trial was the first minimum occurring after onset of the stimulus. Time of maximum constriction (*t*_constr_) was the time elapsed from switching the stimulus (time = 0 s) until reaching the minimum pupil diameter. Mean constriction velocity (*v*_constr_) was calculated by the following formula:

(2) $$v_{\mathrm{constr}}=\frac{\mathrm{Baseline}\;\mathrm{pupil}\;\mathrm{diameter}-PD_{\min}}{t_{\mathrm{constr}}-t_{\mathrm{lat}}}$$

Due to high inter-individual variability in pupil responses following each type of stimulus, we also defined the difference in minimum pupil diameter (*PD*_differ_), which was calculated as the difference between the minimum pupil diameters for each pair of stimuli. For each subject, the mean value of each parameter was calculated as a mean of three separate trials for each stimulus recorded according to the measurement protocol shown in Figure 2A. In addition, the PLR curves were registered for the VIS laser stimuli that elicited a pupil response similar to that for the 800-µW IR laser stimulus for three subjects: P9, P13, and P15. For these subjects, several trials were performed for different VIS laser power levels. After analyzing the obtained images, the trials were repeated three times for selected VIS laser powers eliciting a pupil response similar to that recorded previously for the IR laser.

## Results

### Brightness Adjustment Test

**Figure 4.**
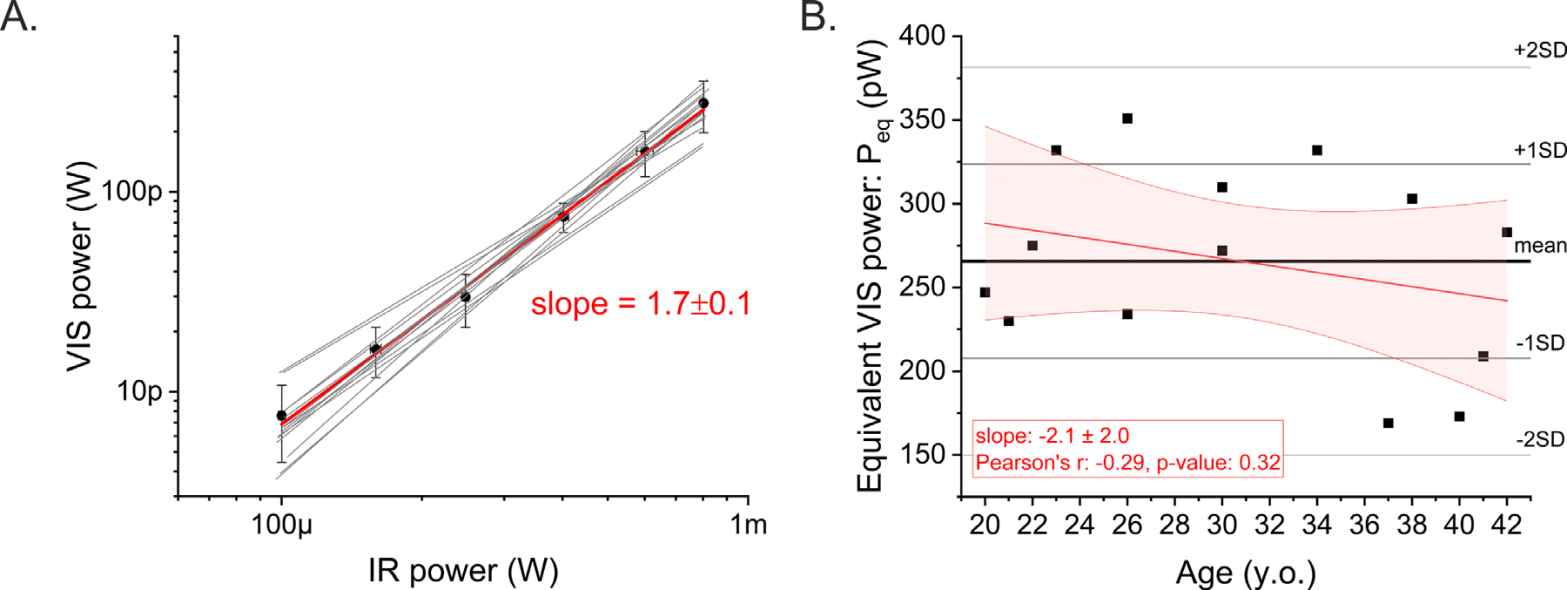
(**A**) Results of brightness adjustment test. *Black squares* indicate the mean power of the VIS stimulus matched to the specified power of the IR stimulus. *Error bars* are 1 SD of the mean. *Horizontal error bars* (barely visible) resulted from the accuracy of the IR beam power setting (with a tolerance of 5%). The *red line* is a linear fit. The *gray* *lines* are linear fits to each subject. (**B**) Equivalent VIS powers (*P*_eq_) as a function of the subject's age. The *solid black line* is a mean value, *gray* *lines* indicate ranges of 1 and 2 SDs. The *red line* is a linear fit with 95% confidence limits indicated by a *red-filled zone*.

The VIS power equivalent to 800 µW of IR stimulus (*P*_eq_) used in consecutive PLR recordings was calculated for each individual from the slope of the log–log fit of VIS power to IR power (Fig. 4A). The individual slopes (1.7 ± 0.3) and the slope calculated for all subjects (1.7 ± 0.1) were significantly greater than 1.0, indicating the nonlinear character of IR stimulus perception, which is consistent with the findings of Ruminski et al.^24^ The individual *P*_eq_ values as a function of the subject's age are shown in Figure 4B. The *P*_eq_ was 266 ± 58 pW (1 SD), and all individual results fell within the range of ±2 SD. For our relatively young study group, there was a weak negative age correlation on determined *P*_eq_ (Pearson's *r* = –0.29), but it was not statistically significant (*P* = 0.31). The slope of the fitted line (–2.1 ± 2.0) was also not significantly different from zero. Therefore, one cannot conclude that there is an age dependence of *P*_eq_ on the basis of these data. All of the subjects perceived the IR stimulus as green; however, some subjects reported the IR beam as more yellowish than the VIS beam,^23^ which might have affected the accuracy of brightness matching.

The repeatability and reliability of the brightness adjustment test were investigated by performing the test twice on the same day on five subjects. The obtained *P*_eq_ values from the two trials are listed in Supplementary Table S1. The between-subject and within-subject SDs calculated based on these results were 81 pW and 25 pW, respectively.^42^ The repeatability coefficient was approximately 70 pW (confidence interval, 40–170).^42^ The reliability of the method, quantified as the intraclass correlation, was estimated to be 0.91.^43^

### Pupillary Light Reflex

**Figure 5.**
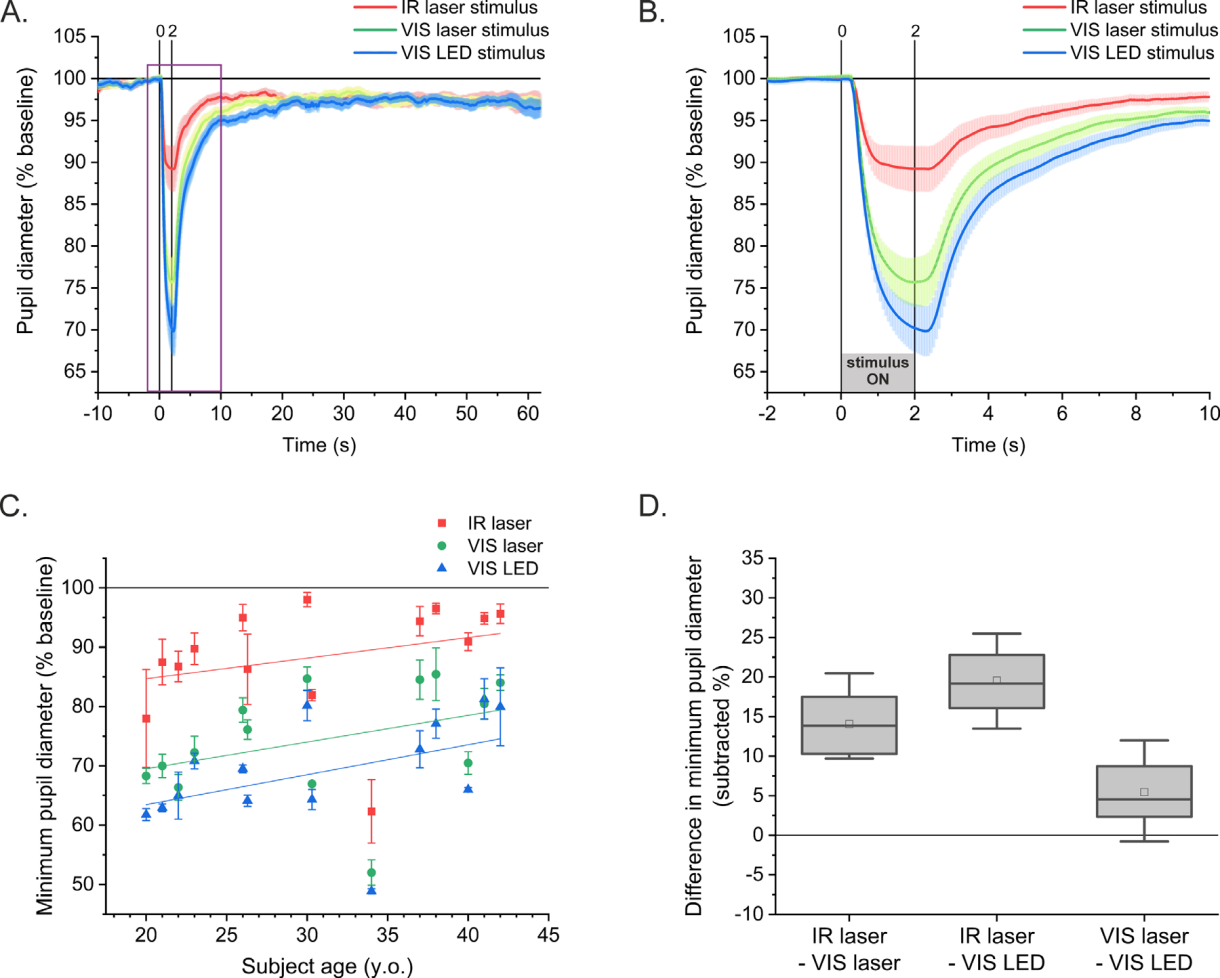
(**A**) Average PLRs of all subjects after three types of stimuli. (**B**) The enlarged area marked by the *purple rectangle* in the graph. The *shaded area* represents the standard error of the mean (SEM). (**C**) Mean minimum pupil diameter of 3 PLR trials as a function of the subject's age; *error bars* indicate 1 SD. *Solid lines* are linear fits. (**D**) Mean differences between pairs of stimuli for all subjects. For the *box chart*, the box is determined by the 25th and 75th percentiles. Mean value, *open square*; median, *solid line*.

The registered PLRs, averaged across all 14 subjects, are shown in Figures 5A and 5B. The mean PLR curves indicate that the IR stimulus caused a generally weaker reaction of the pupil than both VIS stimuli (laser and LED). The VIS laser stimulus also appears to induce a slightly weaker PLR than the VIS LED stimulus. Minimum pupil diameters (Fig. 5C) were significantly smaller for the IR stimulus than for the VIS laser stimulus for 13 of 14 participants (*P* < 0.031, ANOVA with post hoc Tukey's test). For a 20-year-old male subject, the difference was not significant (*P* = 0.11). A significant difference between the IR laser and VIS LED stimuli was found for all subjects (*P* < 0.015). The means of the minimum pupil diameters in response to both VIS stimuli (laser and LED) differed significantly for only six subjects out of 14.

The Grubbs test (Fig. 5C) indicated that the minimum pupil diameter reached after IR laser stimulation of the 34-year-old male was an outlier. Compared with the other participants, this subject's pupil constrictions were the greatest for all types of the stimuli. Because of the neurological origin of the PLR, its nature is highly individual; therefore, we decided not to reject this person's data from the analysis. The minimal pupil diameter of each subject registered for both visible stimuli indicated no significant correlation with VIS equivalent power (*P*_eq_): Pearson's *r* = 0.19 (*P* = 0.51) for the VIS laser and –0.16 (*P* = 0.58) for the LED laser, as shown in Supplementary Figure S3. Minimum pupil diameters registered for all stimulus types did not significantly correlate with the subjects’ refractive errors: correlation coefficient (ρ) = 0.28 (*P* = 0.33) for the IR laser; ρ = 0.22 (*P* = 0.33) for the VIS laser; and ρ = 0.15 (*P* = 0.61) for the LED stimulus. The highest value of correlation for the IR beam may be due to the fact that the two-photon visibility of the IR beam strongly depends on focusing. Lack of significant correlation between refraction and minimal pupil diameter confirms that the correction of refractive errors, although subjective, was sufficient not to substantially affect the obtained results.

The data in Figure 5C indicate a weak positive linear relationship between age and minimum pupil diameter for all of the stimuli. The calculated slopes in percent baseline per year were 0.35 (IR laser, Pearson's *r* = 0.29 with *P* = 0.32), 0.45 (VIS laser, Pearson's *r* = 0.37, with *P* = 0.19), and 0.51 (VIS LED, Pearson's *r* = 0.44 with *P* = 0.11), findings that are in agreement with literature.^21^^,^^44^

Due to the high inter-individual variability of pupil reaction following each type of stimulus, we also considered differences in minimum pupil diameter (*PD*_differ_) between pairs of stimuli (Fig. 5D). The mean difference ± SD calculated across all subjects was 14% ± 4% between the IR and VIS laser stimuli, whereas for the IR laser and VIS LED stimuli, it was 20% ± 4%. The difference between the VIS laser and VIS LED stimuli was 5% ± 4%. Although the latter is small compared with the differences between the IR and VIS stimuli, we note that the visible LED stimulus causes about 6% greater pupillary constriction than scanning laser-based stimulus of the same wavelength. This may be explained by the fact that the LED stimulus was a filled circle as opposed to the spiral laser stimuli, which has a 1° hole inside and thus has a 9% smaller area.

**Figure 6.**
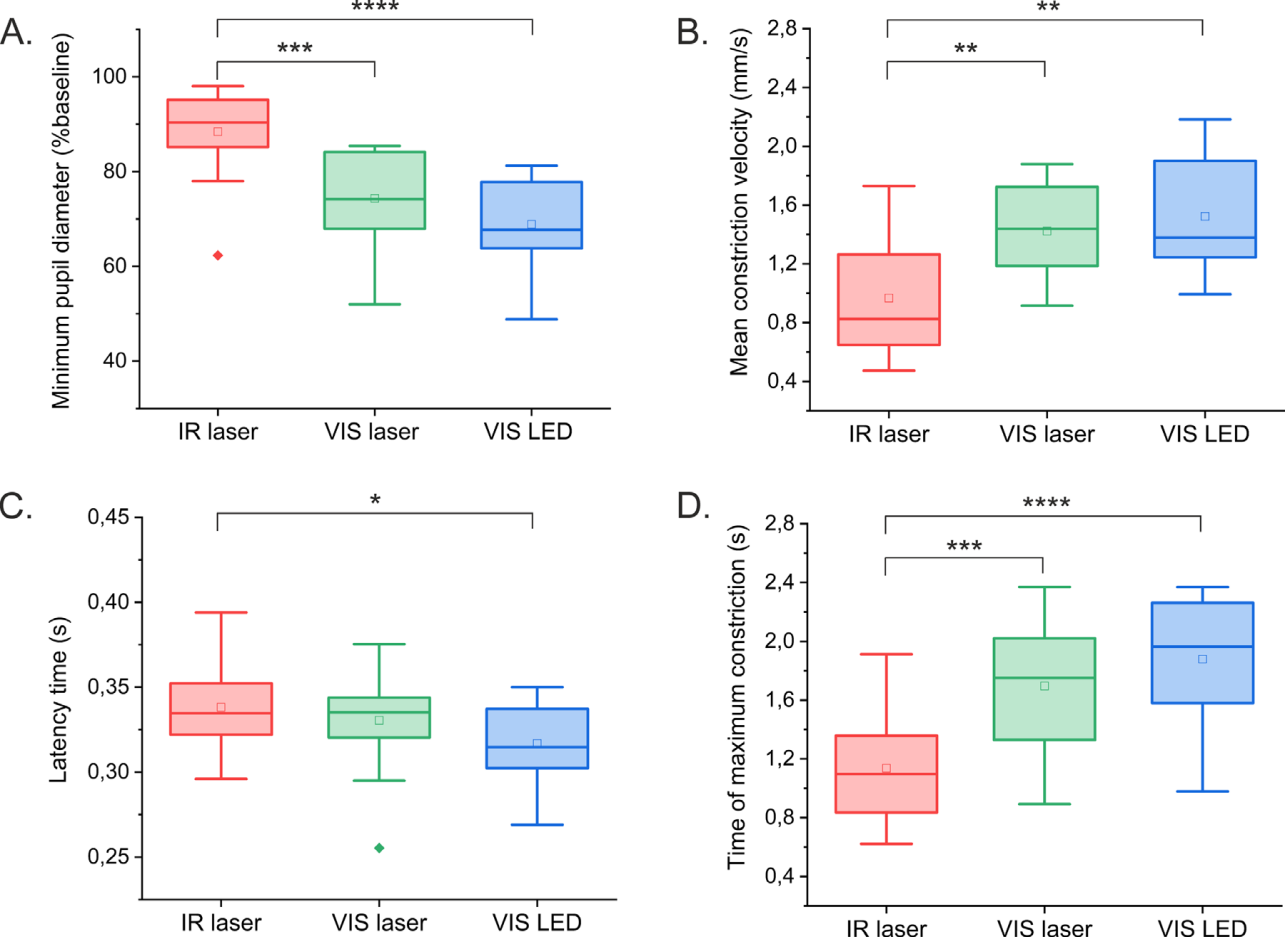
Mean parameters determined from individual PLRs. (**A**) Minimum pupil diameter. (**B**) Mean constriction velocity. (**C**) Latency time. (**D**) Time of maximum constriction. IR laser, *red box*; VIS laser, *green box*; VIS LED, *blue box*. For the *box chart*, the width of the box is limited by the 25th and 75th percentiles. Mean value, *open square*; median, *solid line*. The whiskers are determined by the 5th and 95th percentiles; the outlier is a *bolder point*. The asterisks designate that the means are significantly different: *P ≤ 0.05, **P ≤ 0.01, ***P ≤ 0.001, ****P ≤ 0.0001.

Figure 6 shows four parameters (see Fig. 3) extracted from individual PLR analysis averaged across all participants. The mean minimum pupil diameter (*PD*_min_) (Fig. 6A) registered for the IR stimulus was 88% ± 10%, whereas for both VIS stimuli the values were 74% ± 10% for the laser and 69% ± 9% for the LED. The difference in means was statistically significant for comparison of the IR laser stimulus with both visible stimuli. Although the mean minimum diameter was slightly smaller for the VIS LED than for the VIS laser, the means were not significantly different (*P* = 0.13, ANOVA with post hoc Tukey's test).

Mean constriction velocities (*v*_constr_) (Fig. 6B) were 1.0 ± 0.4 mm/s for the IR laser, 1.4 ± 0.3 mm/s for the VIS laser, and 1.5 ± 0.4 mm/s for the VIS LED. The means were again significantly different for the IR laser and both visible stimuli and did not differ significantly for VIS LED and VIS laser (*P* = 0.46, ANOVA with post hoc Tukey's test). Mean latency times (*t*_lat_) (Fig. 6C) were 340 ± 30 ms for the IR laser, 330 ± 30 ms for the VIS laser, and 320 ± 20 ms for the VIS LED. ANOVA with post hoc Tukey's test indicated significant differences only for the IR laser and VIS LED stimuli (*P* = 0.03). Values of mean times of maximum constriction (*t*_constr_) (Fig. 6D) were 1140 ± 340 ms for the IR laser, 1700 ± 440 ms for the VIS laser, and 1890 ± 440 ms for the VIS LED. Comparisons of means for the IR laser and both visible stimuli showed that they were significantly different, whereas for the VIS laser and VIS LED, they were not (*P* = 0.28, ANOVA with post hoc Tukey's test).

**Figure 7.**
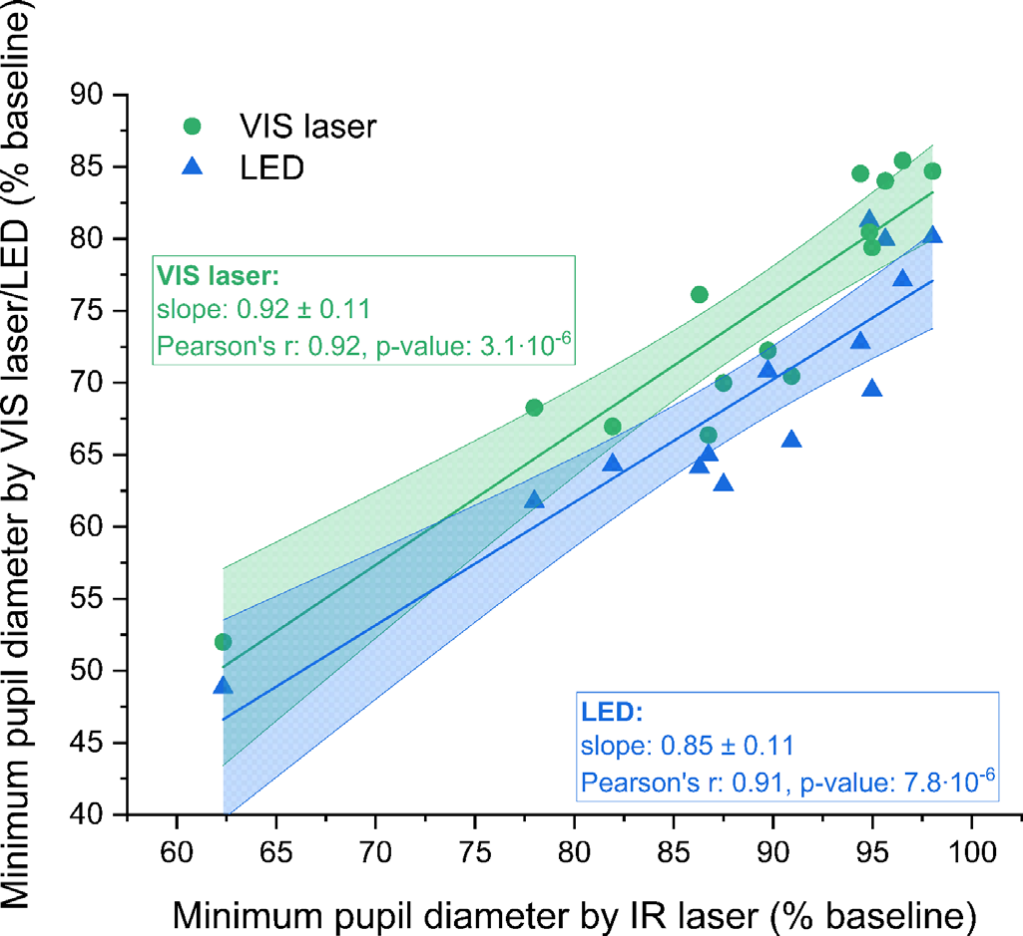
Minimum pupil diameter determined in responses to visible sources plotted as a function of the same parameter in response to IR laser for each subject. Data for the VIS laser and the LED are plotted as *green circles* and *blue triangles*, respectively. The linear function fitted to the VIS laser and LED data is shown with a *green* and *blue solid line*. The 95% confidence intervals for each fit are shown as *semitransparent zones* filled with appropriate colors.

The pupil responses to all types of stimuli can be also compared by examining Figure 7. The minimum pupil diameters caused by both visible stimuli were plotted as a function of minimum diameters triggered by the two-photon laser stimulus for each subject. These data are highly correlated: Pearson's *r* values were 0.92 and 0.91 for the VIS laser and the VIS LED, respectively (*P* = 10^−6^). The strong correlation shows that despite individual differences between subjects, the brightness of the two-photon stimulus was determined properly, as the pupils of subjects with a strong reflex responded strongly to all three types of stimuli, and subjects with a weaker response to the light stimulus had little pupil constriction for all stimulus types. The slopes of the two linear functions fitted to the experimental points are close to 1 (and practically equal to 1 when not including the strongly responsive outlier, which significantly affects the slopes), confirming again the reliability of the brightness adjustment procedure.

**Figure 8.**
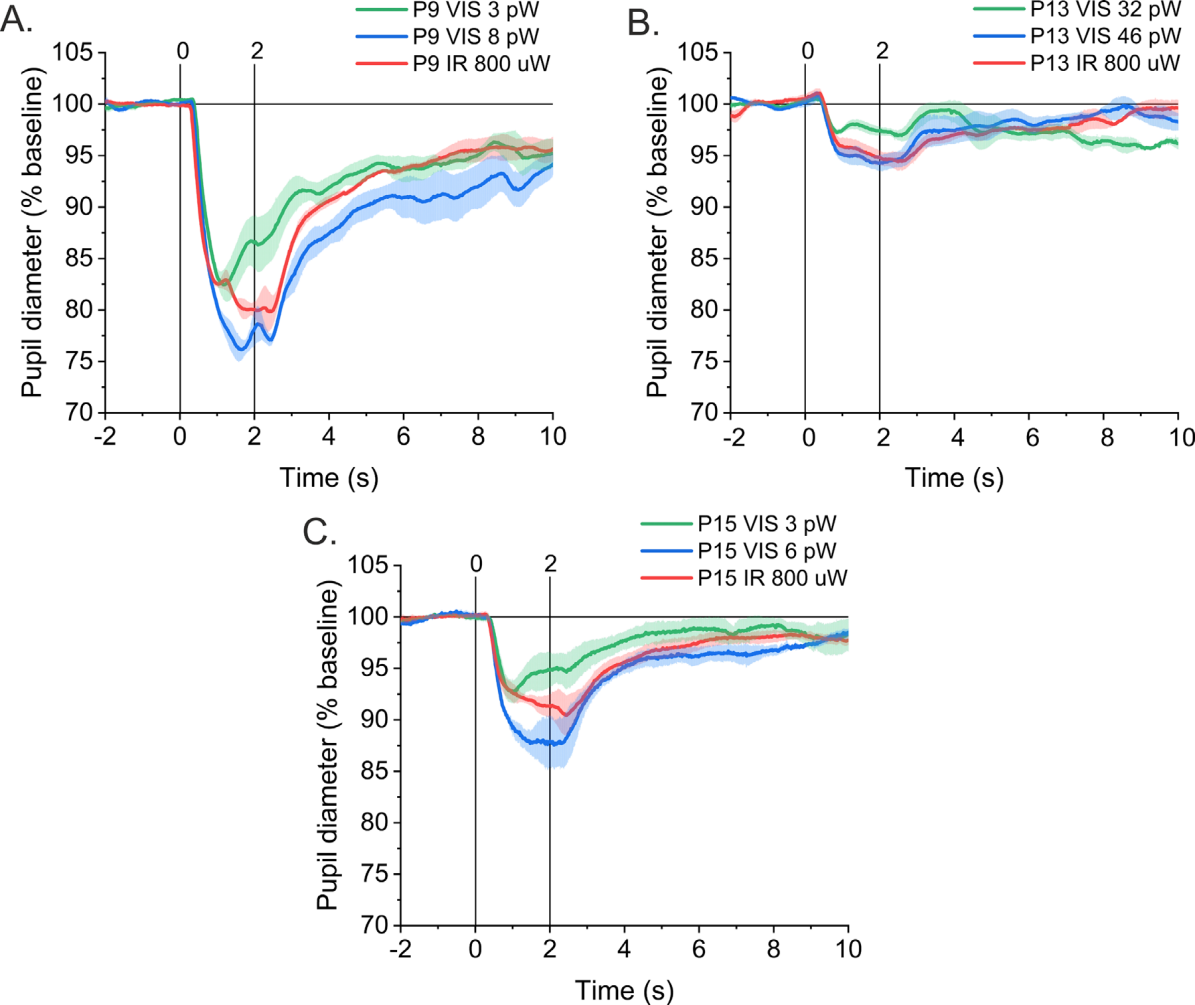
Average PLR curves for the one-photon VIS laser stimulus that elicited a pupil response similar to the 800-µW IR laser two-photon stimulus registered for subjects (**A**) P9, (**B**) P13, and (**C**) P15. VIS equivalent powers estimated by these subjects in the brightness adjustment procedure were 270 pW for P9, 280 pW for P13, and 210 pW for P15. Each *curve* is an average, and *error bars* indicate the standard error of three trials.

The equivalent responses are shown in Figure 8. The powers of the VIS laser eliciting pupil constrictions similar to those of the 800-µW IR laser were 1 to 2 orders of magnitude lower than the *P*_eq_ matched in the brightness adjustment procedure. For two subjects (P9 and P15), these powers were at the level of single picowatts, and for the P13 they were between 30 and 40 pW. The VIS equivalent powers for these subjects were 270 pW and 210 pW for P9 and P15, respectively, and 280 pW for P13. The estimated coefficient of repeatability for the BA analysis was 70 pW (confidence interval, 40–170). The powers of visible stimuli evoking equivalent responses for all three subjects lie outside the range defined by this confidence interval. This result additionally confirms that two-photon stimulation of brightness similar to the one-photon stimulus causes considerably smaller pupil response. The individual mean PLR curves of each subject are presented in Supplementary Figure S4. The raw data have been placed in a public repository (https://doi.org/10.18150/DSF2GN).

## Discussion

The presented results demonstrate that a human pupil constricts in response to a two-photon IR stimulus. However, the reaction is considerably weaker than that caused by the VIS stimulus of the same shape, brightness, and similar color (green). On average, the pupil constricted to 90% of its original size after IR laser stimulus, whereas after both VIS stimuli it constricted to approximately 70%. The mean constriction velocity was also the smallest for the IR stimulus, and the time to maximum constriction was the shortest for the two-photon stimulation. The VIS LED stimulus elicited a slightly stronger response than the VIS laser (Figs. 5A, 5B); however, the differences between the mean values of the four parameters discussed above were not significantly different for both visible stimuli (Fig. 6).

The main difficulty in the present study was an inability to determine the luminosity of the two-photon stimulus. The two-photon absorption cross-sections for visual opsins in vivo have not yet been established, as two-photon psychophysics is an entirely new area that requires further research. The brightness adjustment procedure that we proposed was an attempt to overcome this limitation. The quantified repeatability and reliability of this method (Supplementary Table S1) were further confirmed by the high correlation between minimum pupil diameters evoked by a different type of stimuli, as shown in Figure 7. In Figure 8, we show that for visible stimuli equivalent responses to two-photon stimuli require 1 to 2 orders of magnitude weaker stimulation than found by the brightness adjustment procedure. These findings lead to the conclusion that the pupil constricts for two-photon stimulation of brightness matched to a single-photon stimulus, but as if it is a single-photon stimulus of much lower intensity.

Although the pupil diameter is regulated by ipRGCs that receive extrinsic signals from rods and cones and intrinsic signals originating from melanopsin,^45^ in the case of short-duration and low-intensity stimuli, rod photoreceptors contribute highly to the minimum pupil diameter.^19^,^46^ The weaker stimulation of rods by the two-photon IR beam compared with the VIS beam could lead to a weaker PLR observed in our study. Ruminski et al.^24^ provided experimental evidence indicating that rods are stimulated to a lesser extent by the two-photon laser beam of 1040 nm than 520 nm, although both wavelengths are perceived as green. Dark adaptation curves obtained for these two stimuli were different: the rod–cone interval was significantly smaller, and the rod–cone break occurred later for the two-photon 1040 nm stimulus compared with the one-photon 520-nm stimulus. Hypothesized explanations were spectral differences in two-photon absorption cross-sections of cone and rod opsins or waveguiding properties of cones resulting in higher light flux density at the cone outer segment.^24^ The exact explanation for this observation still requires further study.

These hypothetical differences in the degree of two-photon stimulation between cones and rods compared with normal vision might also explain the difference in hue between IR and VIS laser stimuli reported by some of our subjects during the brightness adjustment test. It is known that rods also contribute to color perception in mesopic and scotopic vision, which causes the change in perceived hue compared with photopic conditions.^47^

The direct ipRGC stimulation in our experiment was unlikely; the retinal exposure for visible light sources (520 nm) was 9.26 log photons/(cm^2^⋅s). The ipRGC threshold is ∼11 log photons/(cm^2^⋅s), and this value was found for the wavelength 480 nm,^48^ close to the maximum spectral sensitivity of ipRGC melanopsin (482 nm).^49^ The ipRGC activation threshold for 520 nm is then larger than for 480 nm. The PLRs showing ipRGC melanopsin activation were typically induced by the blue light of retinal irradiance above 13 log photons/(cm^2^⋅s).^18^ The ipRGCs are also not activated by a visible red light—the spectral sensitivity of melanopsin at 600 nm is 2.5 orders of magnitude lower than in the maximum.^20^ Therefore, one-photon stimulation of these cells by our IR laser beam probably did not occur, although the irradiation level for the 1040-nm wavelength was considerably higher than for 520 nm. The lack of ipRGC stimulation is additionally confirmed by the lack of a post-illumination pupil response^20^ in the registered curves (Fig. 5A). Some of the individual PLR curves presented in Supplementary Figure S4A exhibit features of pupil escape, which serves as another confirmation that ipRGCs are not stimulated by IR lasers and absorption at photoreceptors is low.^19^

An additional factor explaining the weaker IR response may be that the two-photon stimulation of photoreceptors takes place only in the focal region of the laser beam, where the light intensity is sufficiently high for the two-photon absorption. In contrast to both visible stimuli, the scattered IR light is not perceived and probably had no significant effect on the recorded pupil responses. This assumption was confirmed by the lack of pupil reflex for the IR stimulus below the visibility threshold as shown in Supplementary Figure S5. Eventually, fewer photoreceptors are involved in the light perception of two-photon stimulus than in the case of the one-photon stimuli. It should be noted here that the amount of stray light produced by the subjects’ eyes for stimulation by a 1040-nm beam of power 800 µW remains unknown, and measuring it lies beyond the scope of this work. The existing methods of measuring stray light rely on measuring the visual thresholds/equivalent illumination^50^^,^^51^ and thus require that stray light be perceived by the subject.

In this work, for the first time to the best of our knowledge, we have demonstrated that a human pupil constricts in response to a pulsed infrared stimulus perceived due to two-photon stimulation. This stimulation causes considerably weaker reactions than one-photon stimuli subjectively adjusted to the same brightness. We hypothesize that this observation can be explained by two factors. First, the two-photon stimulation of rods is weaker as compared with one-photon stimulation of the same brightness. Second, the scattered light is not perceived by two-photon vision, which reduces the number of stimulated photoreceptors. Our results are important for understanding the fundamentals of two-photon–based light perception and the development of ophthalmological devices using pulsed infrared laser beams that may be perceived due to two-photon vision.

## Supplementary Material

Supplement 1
