# Supplementary material for: Pupillary Light Reflex Induced by Two-Photon Vision
Source: Invest Ophthalmol Vis Sci. 2021 Dec 22;62(15):23. doi: 10.1167/iovs.62.15.23 (PMC8711009; doi:10.1167/iovs.62.15.23)
Supplement: Supplement 1 [file iovs-62-15-23_s001.pdf]

Supplementary material

A. Zielińska, P. Ciąćka, M. Szkulmowski and K. Komar; Pupillary Light Reflex induced by two-photon vision

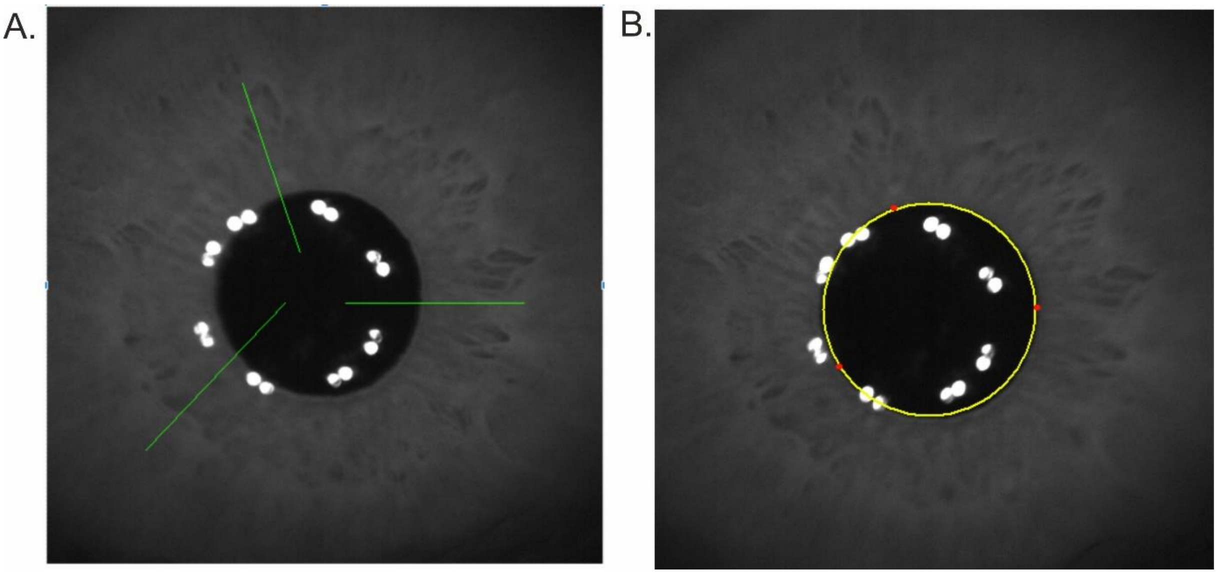

Figure S1. (A) Exemplary pupil image with three green lines indicated by the operator at the beginning of images series. **(B)** The same image with the edges of the pupil found by the software along the green lines and with a fitted yellow circle based on those points that define the pupil.

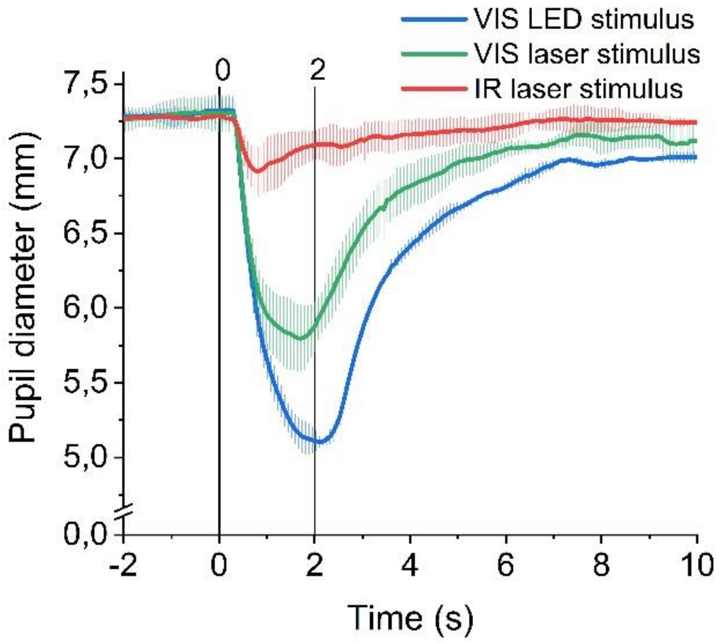

Figure S2. Average PLR curves of a representative subject (P2) with the ordinate axis scale expressed in mm – supplemental to Fig. 3C. Each curve is an average and error bars indicate the standard deviation of three trials.

Table S1. The repeatability and reliability of brightness adjustment procedure.

**Subject**	**P1**	**P4**	**P9**	**P12**	**P13**
**Trial 1 P_{eq} [pW]**	272	356	216	154	220
**Trial 2 P_{eq} [pW]**	224	395	172	160	239
**Subject's means [pW]**	**248**	**375.5**	**194**	**157**	**229.5**
**Total mean [pW]**	**240.8**				

	**Sum of squares [pW²]**	**Degrees of freedom**	**Mean square (MS) [pW²]**	**Standard deviation (SD) [pW]** [1]
**Within subjects (WS)**	3079	5	MS_{WS} = 615.8	$SD_{WS} = \sqrt{MS_{WS}} \cong 25$
**Between subjects (BS)**	55072.6	4	MS_{BS} = 13768.15	$SD_{BS} = \sqrt{\frac{MS_{BS}-MS_{WS}}{2}} \cong 81$
**Repeatability coefficient [pW]** [1]	RC = $2.77 \cdot SD_{WS} \cong 70$	**Confidence intervals [pW]** [1]	CI = $\left(\sqrt{\frac{5 \cdot MS_{WS}}{\chi_5^2(0.025)}}, \sqrt{\frac{5 \cdot MS_{WS}}{\chi_5^2(0.975)}}\right) \cong (40, 170)$	
**Reliability (Intraclass correlation)** [2]	ICC = $\frac{{SD_{BS}}^2}{{SD_{BS}}^2 + {SD_{WS}}^2} = 0.91$			

1. Barnhart, Huiman X., and Daniel P. Barboriak. "Applications of the repeatability of quantitative imaging biomarkers: a review of statistical analysis of repeat data sets." *Translational oncology* 2.4 (2009): 231-235.
2. Bartlett, J. W., and Chris Frost. "Reliability, repeatability and reproducibility: analysis of measurement errors in continuous variables." *Ultrasound in Obstetrics and Gynecology: The Official Journal of the International Society of Ultrasound in Obstetrics and Gynecology* 31.4 (2008): 466-475.

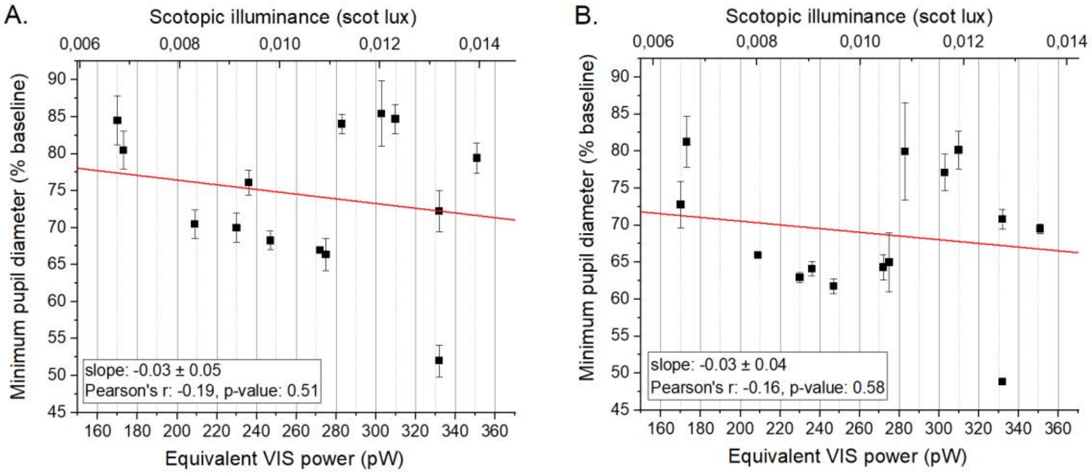

Figure S3. The minimum pupil diameter of each subject plotted as a function of VIS equivalent power/scotopic illuminance determined in brightness adjustment procedure for: **(A)** the VIS laser stimulus and **(B)** the LED stimulus. Error bars indicate one standard deviation.

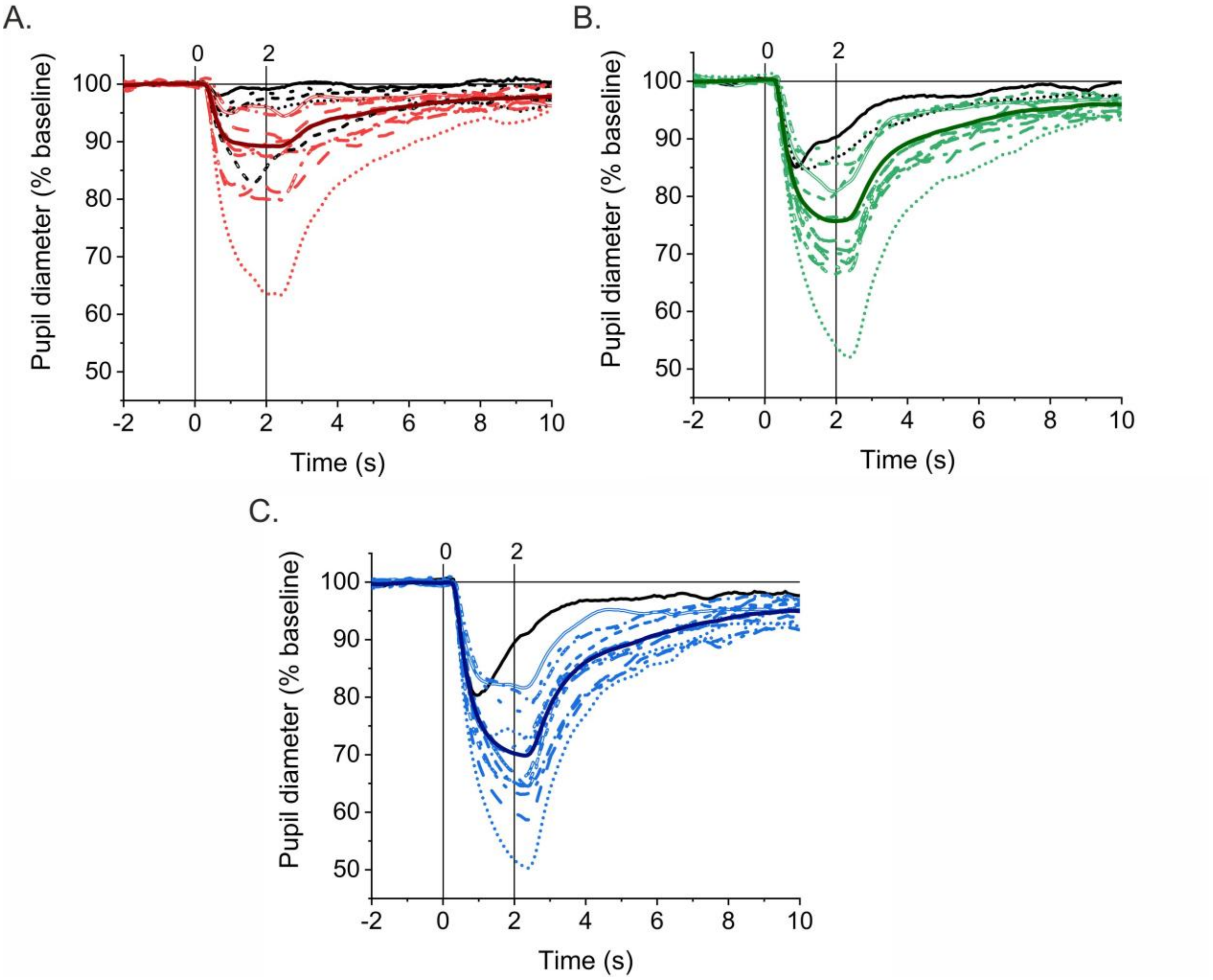

Figure S4. Individual PLR curves registered for: **(A)** IR laser stimulus, **(B)** VIS laser stimulus and **(C)** VIS LED stimulus. Each curve is the average from 3 trials, the line style is assigned to the same subject in all plots. The trials exhibiting characteristics of pupil escape are plotted with black. Bolded solid line is a mean across all subjects. Individual raw data are also available at public repository: https://doi.org/10.18150/DSF2GN

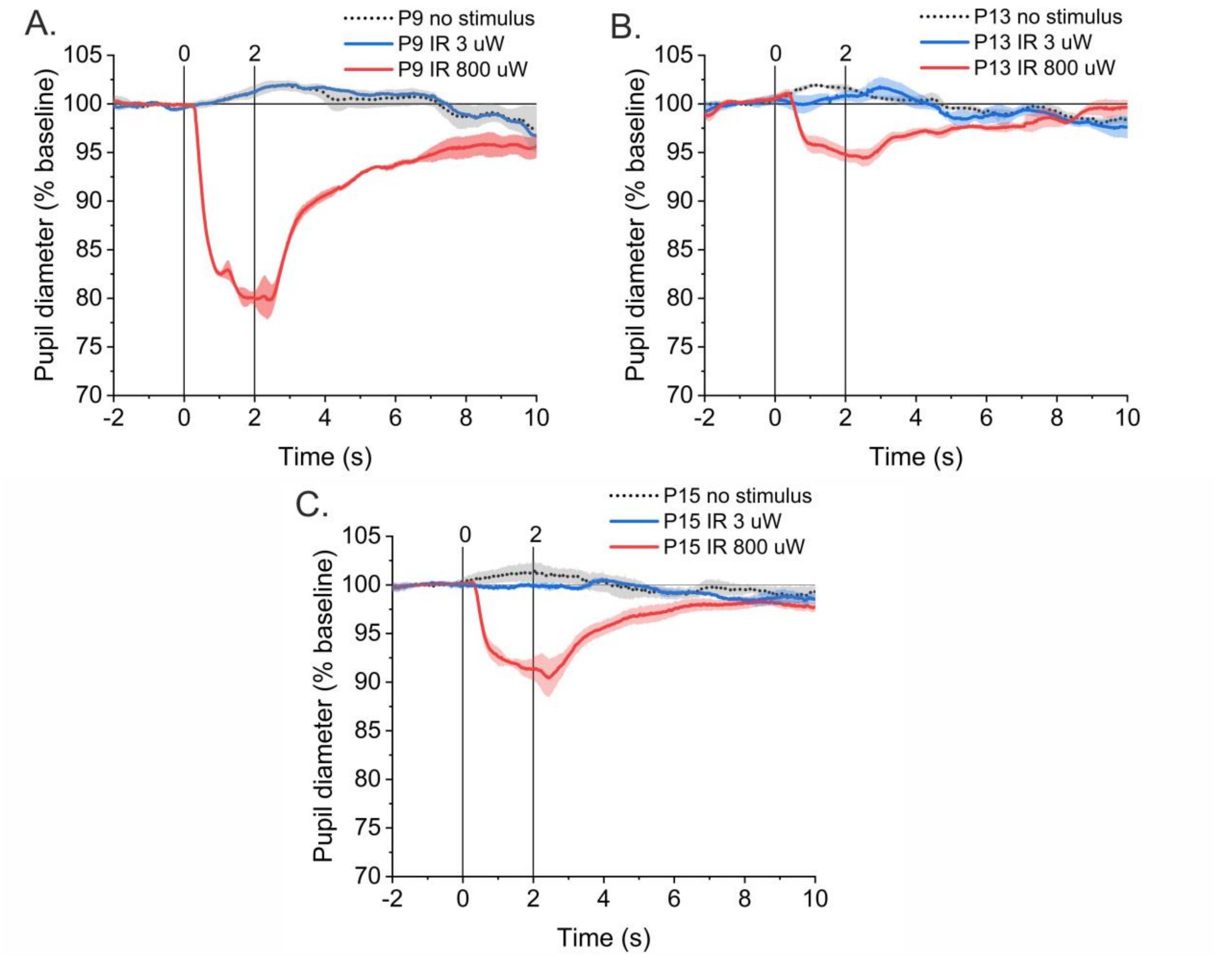

Figure S5. Average PLR curves for IR stimuli of 800 μW (red) and 3 μW (blue) for subjects: **(A)** P9, **(B)** P13 and **(C)** P15. The stimulus below the visibility threshold (3 μW) does not elicit pupil reaction. The black dotted lines show trials with IR laser-off for comparison. Each curve is an average, and error bars indicate the standard error of three trials.
